# Pregnant women with COVID-19 and risk of adverse birth outcomes and maternal-fetal vertical transmission: a population-based cohort study in Wuhan, China

**DOI:** 10.1186/s12916-020-01798-1

**Published:** 2020-10-19

**Authors:** Rong Yang, Hui Mei, Tongzhang Zheng, Qiang Fu, Yiming Zhang, Stephen Buka, Xinan Yao, Zezhong Tang, Xichi Zhang, Lin Qiu, Yaqi Zhang, Jieqiong Zhou, Jiangxia Cao, Youjie Wang, Aifen Zhou

**Affiliations:** 1 Wuhan Children’s Hospital (Wuhan Maternal and Child Healthcare Hospital), Tongji Medical College, Huazhong University of Science & Technology, 100 Xianggang Road, Wuhan, 430030 China; 2 School of Public Health, Brown University, Providence, RI 02903 USA; 3 Department of Epidemiology and Biostatistics, College for Public Health and Social Justice, Saint Louis University, 3545 Lafayette Ave., St. Louis, MO 63104 USA; 4 Krannert School of Management, Purdue University, 475 Stadium Mall Drive, West Lafayette, IN 47906-2050 USA; 5 Department of Pediatrics, Peking University First Hospital, 8 Xishiku Street, Beijing, 100034 China; 6 Rollins School of Public Health, Emory University, 1518 Clifton Rd, NE, Atlanta, GA 30322 USA; 7 Department of Maternal and Child Health, School of Public Health, Tongji Medical College, Huazhong University of Science &Technology, 13 Hangkong Road, Wuhan, 430030 China

**Keywords:** COVID-19, Birth outcome, Maternal-fetal vertical transmission

## Abstract

**Background:**

The coronavirus disease 2019 (COVID-19) outbreak is evolving rapidly worldwide. However, little is known about the association between pregnant women with COVID-19 and the risk of adverse birth outcomes.

**Method:**

We conducted a retrospective cohort study based on the Maternal and Child Health Information System (MCHIMS) of Wuhan, China. All pregnant women with singleton live birth recorded by the system between January 13 and March 18, 2020, were included. The adverse birth outcomes were preterm birth, low birth weight, neonatal asphyxia, premature rupture of membrane (PROM), and cesarean section delivery. Multivariate logistic regression was used to evaluate the associations between maternal COVID-19 diagnosis and adverse birth outcomes.

**Results:**

Out of 11,078 pregnant women, 65 were confirmed with coronavirus disease 2019 (COVID-19). No deaths occurred from these confirmed cases or their newborns. Compared to pregnant women without COVID-19, pregnant women with a confirmed COVID-19 diagnosis had an increased risk of preterm birth (OR 3.34, 95% CI 1.60–7.00) and cesarean section (OR 3.63, 95% CI 1.95–6.76). There was no statistical difference in low birth weight, neonatal asphyxia, and PROM between the mothers with and without COVID-19. Among these newborns that were born to mothers with confirmed COVID-19, none was tested severe acute respiratory syndrome coronavirus 2 (SARS-CoV-2) positive or had abnormal CT results. Only one had diarrhea and three had a fever.

**Conclusions:**

This population-based cohort study suggests that COVID-19 during the later pregnancy is associated with an increased risk of adverse birth outcomes, including iatrogenic preterm birth and cesarean section delivery. Our data provide little evidence for maternal-fetal vertical transmission of SARS-CoV-2. It is important to monitor the long-term health effects of SARS-CoV-2 infection on pregnant women and their children.

## Background

The outbreak of SARS-CoV-2 infection has become a global epidemic threat since the end of 2019 [1–4]. As of April 14, the cumulative number of confirmed COVID-19 cases had reached 1,930,000 with 120,000 (6%) deaths worldwide. The world is now launching a forceful, focused campaign to eradicate COVID-19. Wuhan, the capital of Hubei province where COVID-19 was first reported, was the hardest-hit Chinese city and accounts for 60% of confirmed cases and 83% of COVID-19 deaths in China. SARS-CoV-2 is a new virus that results in a spectrum of illness ranging from asymptomatic to severe acute respiratory distress syndrome and death [2, 3, 5]. The consequences of infection with SARS-CoV-2 among pregnant women are currently uncertain. Several hundreds of pregnant women in Hubei province were infected with SARS-CoV-2 based on infectious disease surveillance systems in Hubei province, China. While several case series studies have analyzed the clinical symptoms and prognosis of COVID-19 cases, no population-based study so far has been conducted to examine the relationship between SARS-CoV-2 infection and adverse birth outcomes.

Several case series with small sample sizes have examined the potential for in utero vertical transmission of SARS-CoV-2 in infected pregnant women in China. In a report involving two pregnant women with confirmed COVID-19, the two newborns were reported to be negative for SARS-CoV-2 tests [6]. A study involving 9 confirmed pregnant women with COVID-19 in Wuhan found no evidence of in utero vertical transmission of SARS-CoV-2 among their newborns [7]. A study involving 6 newborns born to mothers with confirmed COVID-19 also reported negative SARS-CoV-2 tests while virus-specific antibodies IgM and IgG were detected in 2 of the newborns, suggesting a possibility of in utero SARS-CoV-2 infection [8]. A recent single case study from Wuhan also suggests the potential in utero infection of the neonate [9].

Large population-based studies are urgently needed to evaluate if SARS-CoV-2 infection during pregnancy could affect pregnancy outcomes and result in utero vertical transmission. Such data would provide key information for the protection of women and children. This population-based cohort study in Wuhan for the first time evaluated the relationship between SARS-CoV-2 infection during later pregnancy and risk of adverse birth outcomes including preterm birth, low birth weight, PROM, neonatal asphyxia, and cesarean section. It also investigated the potential in utero vertical transmission at the population level.

## Methods

### Sample

**Fig. 1:**
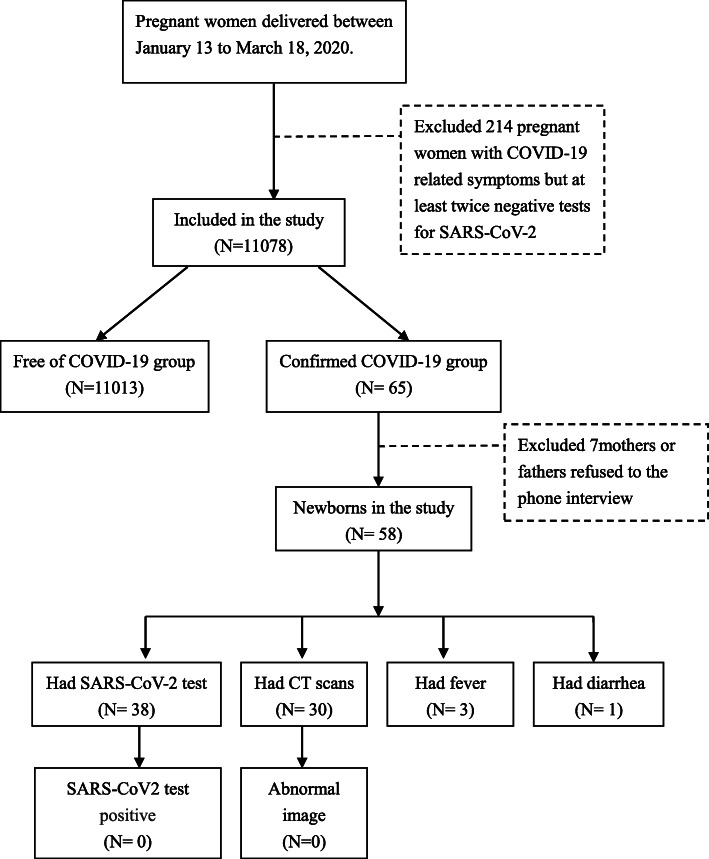
Flow chart of study participants

This population-based cohort study was conducted in Wuhan, the first reported city of the outbreak of COVID-19 in China. We used the Maternal and Child Health Information Management System of Wuhan (MCHIMS) to identify the study population. The MCHIMS is used to monitor maternal and children’s health by collecting information for all pregnant women and their children in the Wuhan metropolitan area. During the outbreak of COVID-19 in Wuhan, in addition to routine prenatal care data from clinical and laboratory examinations and socio-demographic information, the MCHIMS also recorded the COVID-19 diagnosis for pregnant women as part of the high-risk pregnancy surveillance during the COVID-19 pandemic in Wuhan. The pregnant women of Wuhan residents satisfying the following conditions will be included in the study: (1) gave a single live birth between January 13 and March 18, 2020 (that is, between the date of the first recorded COVID-19 case of pregnant women and the last date with available data for the study from the MCHIMS system), and (2) registered by the MCHIMS with either confirmed COVID-19 or free of COVID-19. We excluded the 214 pregnant women with COVID-19-related symptoms but at least twice tests of a negative result of SARS-CoV-2 detection. Finally, a total of 11,078 pregnant women satisfied the study criteria and were included in this study, 65 of them were recorded as confirmed cases of COVID-19. Among 11,013 women without a diagnosis of COVID-19 pregnant women, 4778 were admitted before February 3, 2020. The flow chart for the recruitment of study subjects is presented in Fig. 1.

### Diagnosis of COVID-19

The study population was classified into confirmed COVID-19 group and free COVID-19 group. The diagnosis of COVID-19 was based on the clinical diagnostic criteria of the New Corona Virus Infected Pneumonia Diagnosis and Treatment Plan issued by the Chinese National Health and Health Commission in 2020 [10]. The confirmed cases of COVID-19 had taken at least twice tests for SARS-CoV-2, which used real-time RT-PCR based on a pharyngeal swab. During the outbreak, all pregnant women in Wuhan were under increased surveillance for COVID-19 and given top priority for SARS-CoV-2 testing and hospitalization. Due to the lack of available tests during the early phase of the pandemic (before February 4, 2020), only the pregnant women developing the symptoms like fever and cough or having abnormal computed tomography (CT) scan were tested for SARS-CoV-2. Since February 4, 2020, all pregnant women giving birth in hospitals were screened for the SARS-CoV-2. Pregnant women without COVID-19-related signs or symptoms were tested for the virus only once, while pregnant women with COVID-19-related signs or symptoms were tested for at least two times if the first detection was negative. Because of the assumption from the severe acute respiratory syndrome (SARS) studies that there is no vertical transmission, all the newborns born to the infected mothers were separated from their mother immediately after delivery and were brought home unless medical observation or treatment was needed. To obtain information on COVID-19 diagnosis or SARS-CoV-2 infection status for the newborns born to these mothers with confirmed COVID-19, the study obstetricians at Wuhan Children’s Hospital made follow-up phone calls. Mothers diagnosed with COVID-19 were directly transferred to specialty hospitals after delivery for treatment. After successful treatment, they were removed to facilities for quarantine for 14 days and these follow-up calls were made to their home usually after their discharge. All participants provided oral informed consent before the telephone interview.

### Maternal and newborn variables

The information in the phone interviews was typically provided by the mothers, rarely by fathers. Maternal information abstracted from the MCHIMS includes age, education, employment, gestational age, gravidity, parity, pregnancy-induced hypertension, gestational diabetes mellitus, PROM, and SARS-CoV-2 infection. For the newborns, the abstracted information included sex, gestational week, preterm birth, birth weight, and neonatal asphyxia. Both maternal and newborn information was input based on the medical records by the health professionals in the delivery hospital. Primary outcomes for the newborns were preterm birth (< 37 weeks of pregnancy), low birth weight (< 2500), PROM (defined as rupture of membrane before the onset of labor), and neonatal asphyxia (defined as 1 min Apgar score ≤ 7 and umbilical arterial blood gas pH < 7.15 [11]).

### Statistical analyses

Study population characteristics are presented by proportion for categorical variables. Univariate chi-square analyses were conducted to evaluate rates of birth outcomes by comparing the two groups of mothers with or without confirmed COVID-19. Multivariate logistic regression models were used to evaluate the associations between maternal COVID-19 status and adverse birth outcomes, adjusted for potential confounding variables. The following variables were included in the final models: maternal age (14–24, 25–34, 35–54), occupation (employed, housewives, part-time), education (bachelor’s degree or above, high school, vocation degree, middle school or below), gravidity (1, 2, 3–10), parity (1, 2, 3–5), gestational hypertension (yes, no), preeclampsia (yes, no), gestational diabetes mellitus (yes, no), and PROM (yes, no). All analyses were performed using SAS version 9.4 (SAS Institute, Inc., Cary, North Carolina). The study was approved by the Human Ethics Committees at Wuhan Children’s Hospital.

## Results

Of the 11,078 pregnant women with singleton live births during the study period, 65 (0.57%) were diagnosed with COVID-19. Table 1 shows the demographic variables and pregnancy complications for these 11,078 pregnant women, and there were few differences between these pregnant women based on their COVID-19 status. Confirmed cases had higher educational attainment than the rest of the sample, with modest occupational differences as well.

**Table 1:** Distribution of the characteristics of the study population by maternal COVID-19 status in Wuhan, China

Variables	COVID-19 status		*P*
	Free	Confirmed	
**Age range, years**			.60
< 25	805 (7)	4 (6)	
25–34	8610 (78)	54 (83)	
≥ 35	1598 (15)	7 (11)	
**Education levels**			< 0.001
Bachelor’s degree or above	3179 (29)	42 (65)	
High school	1634 (15)	5 (7)	
Vocation degree	1952 (18)	7 (11)	
Middle school or below	2359 (21)	8 (12)	
Missing	1889 (17)	3 (5)	
**Occupation**			.04
Employed	3874 (35)	33 (51)	
Housewives	4001 (36)	20 (31)	
Part-time	3138 (29)	12 (18)	
**Gravidity**			.99
1	4658 (42)	27 (42)	
2	3182 (29)	19 (29)	
≥ 3	3173 (29)	19 (29)	
**Parity**			.36
1	6425 (58)	41 (63)	
2	4318 (39)	21 (32)	
≥ 3	27 (3)	3 (5)	
**History of abortion**			.57
0	6692 (61)	37 (57)	
1–2	3794 (34)	23 (35)	
≥ 3	527 (5)	5 (8)	
**Gestational hypertension**			.44
Yes	325 (3)	3 (5)	
No	10,688 (97)	62 (95)	
**Preeclampsia**			.39
Yes	83 (1)	1 (1)	
No	10,930 (99)	64 (99)	
**Gestational diabetes mellitus**			.07
Yes	1207 (11)	3 (5)	
No	9806 (89)	62 (95)	

Table 2 shows that the mothers with confirmed COVID-19 had a significantly higher rate of preterm birth and cesarean section. All preterm babies born to infected mothers in the present study were iatrogenic preterm birth. No significant differences in neonatal asphyxia, low birth weight, and PROM were observed between the two groups.

**Table 2:** The birth outcomes of the newborns by maternal COVID-19 status in Wuhan, China

Variables	COVID-19 status		*P*
	Free	Confirmed	
**Sex**			.90
Male	5880 (53)	36 (55)	
Female	5124 (47)	29 (45)	
Unknown	9 (0)	0	
**Asphyxia**			.33
Yes	158 (1)	2 (3)	
No	10,855 (99)	63 (97)	
**PROM**			.15
Yes	1248 (11)	4 (6)	
No	9765 (89)	61 (94)	
**Preterm birth**			.01
Yes	579 (5)	9 (14)	
No	10,434 (95)	56 (86)	
**Delivery mode**			< 0.001
Vaginal delivery	4993 (45)	13 (20)	
Cesarean section	6020 (55)	52 (80)	

**Fig. 2:**
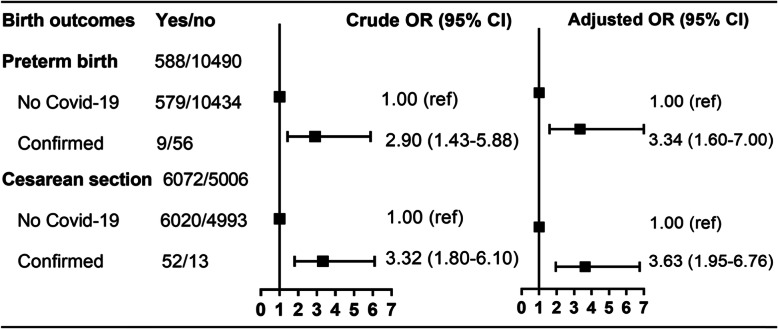
Risk of adverse birth outcomes by maternal COVID-19 status in Wuhan, China. Adjusted for maternal age (14–24, 25–34, 35–54), occupation (employed, housewives, part-time), education (bachelor’s degree or above, high school, vocation degree, middle school or below), gravidity (1, 2, 3–10), parity (1, 2, 3–5), gestational hypertension (yes, no), preeclampsia (yes, no), gestational diabetes mellitus (yes, no), and premature rupture of membranes (yes, no)

Figure 2 shows the multivariate logistic regression results for the associations between maternal COVID-19 status and the risk of adverse birth outcomes. Compared to mothers without COVID-19, mothers with confirmed COVID-19 had an adjusted OR of 3.34 (95% CI 1.60, 7.00) for preterm birth and an adjusted OR of 3.63 (95% CI 1.95, 6.76) for receiving cesarean section.

Table 3 presents the association between maternal COVID-19 diagnosis and preterm birth from mothers with cesarean section delivery. Compared to mothers without COVID-19, mothers with confirmed COVID-19 had an adjusted OR of 3.71 (95% CI 1.70, 8.03) for preterm birth among mothers with cesarean section delivery.

**Table 3:** The association between maternal COVID-19 diagnosis and preterm births from mothers receiving the cesarean section

COVID-19	Preterm birth		OR^†^ (95% CI)	OR^‡^ (95% CI)
	Yes	No		
Free	363	5657	1.00	1.00
Confirmed	9	43	3.26 (1.58, 6.74)	3.71 (1.70, 8.03)

^†^Crude ORs

^‡^Adjusted for maternal age (14–24, 25–34, 35–54), occupation (employed, housewives, part-time), education (bachelor’s degree or above, high school, vocation degree, middle school or below), gravidity (1, 2, 3–10), parity (1, 2, 3–5), gestational hypertension (yes, no), preeclampsia (yes, no), gestational diabetes mellitus (yes, no), and premature rupture of membranes (yes, no)

We reached 58 parents of the 65 confirmed maternal COVID-19 cases (89%) through phone calls by study obstetricians to determine the newborns’ COVID-19 and/or SARS-CoV-2 infection status. As shown in Table 4, out of 58 newborns born to mothers with confirmed COVID-19, 38 newborns had the SARS-CoV-2 test and none of the newborns tested positive for SARS-CoV-2. For those who had CT scans, none of the 30 newborns born to mothers with confirmed COVID-19 was reported to have abnormal CT scan images. Three of the 58 newborns born to the mothers with confirmed COVID-19 reported to have fever, and 1 of them had diarrhea.

**Table 4:** The clinical manifestation and SARS-CoV2 test results for the 58 newborns born to mothers with confirmed COVID-19 in Wuhan, China

Test results or symptoms	Newborns (*n* = 58)
SARS-CoV2 test positive	0/38
Abnormal image of chest CT	0/30
Diarrhea	1/58
Fever	3/58

## Discussion

Using population-based data for 11,078 pregnant women and their singleton live births in Wuhan city, we for the first time investigated if SARS-CoV-2 infection affects pregnancy outcomes and evidence for potential vertical transmission. Our study results showed that pregnant women with confirmed COVID-19 had an increased risk of adverse birth outcomes including preterm birth, and delivery with cesarean section compared to pregnant women without COVID-19. We also found no strong evidence suggesting a vertical maternal-fetal transmission of SARS-CoV-2.

Early studies have shown that physiologic and immunologic changes during pregnancy might increase the risk for pregnant women to be infected with respiratory viruses such as influenza [12, 13]. It has been reported that pregnant women are more susceptible to be infected, develop more severe complications of the disease, and have higher mortality compared to the non-pregnant population [14]. However, the infection rate of SARS-CoV-2 among pregnant women (0.57%) in the present study was comparable to that (0.50%) in the general population in Wuhan. Unlike several hospital-based studies with small sample sizes that have shown that SARS infection increases morbidity and mortality of pregnant women [15], no deaths were reported among the confirmed COVID-19 cases from the current sample of 11,078 pregnant women.

In our study, the confirmed COVID-19 mothers had higher educational attainment than mothers without the disease, with modest occupational differences as well. In China, people with higher education are more likely employed and, due to the severe traffic jam experienced in Wuhan, working people frequently used public transportation to commute to work, which increases opportunities to be exposed to the virus. Increased interpersonal contacts at the workplace further increase their risk of infection.

Our study demonstrated that pregnant women with COVID-19 were more likely to have preterm birth babies. Considering all preterm babies were born to infected mothers were iatrogenic preterm birth due to intrauterine fetal distress, we examined the possibility that the elevated risk for preterm birth resulted from higher rates of elective and early cesarean sections (Table 3), the positive association still exists among mothers with cesarean section. Previous studies have also shown that SARS and Middle East respiratory syndrome (MERS) infections are related to preterm birth, intensive care treatment for newborns, and even perinatal death [15]. A higher rate of cesarean section was found among the infected mothers in the present study; the odds of cesarean births were three times or greater among women with COVID-19 compared to those without COVID-19. However, only when there were indications posed by SARS-CoV-2 infection to pregnant women or fetuses, such as maternal breathlessness and related complications as well as fetal intrauterine distress, cesarean sections were performed as needed. Thus, those symptoms of COVID-19 have contributed to the high rate of cesarean section among the infected mothers.

It is of great concern if there is a maternal-fetal vertical transmission after SARS-CoV-2 infection. In the present study, 38 newborns born to 65 mothers with confirmed COVID-19 had tested for SARS-CoV-2, and all of them had negative test results. Similarly, several hospital-based small case series studies conducted in Wuhan also do not support vertical transmission [16, 17]. The lack of maternal-fetal transmission was also reported in early studies of SARS and MERS infection in pregnant women [15]. However, several recent case series have suggested a possibility of vertical transmission of SARS-CoV-2, including a recently reported single case study from Wuhan that shows a neonate born to a mother with COVID-19 had elevated IgM antibody level 2 h after birth [8, 9]. Since IgM antibodies are not transferred to the fetus via the placenta [18], and usually do not appear until 3 to 7 days after infection, the observation appears to support that the neonate was infected in utero. However, the results from 5 RT-PCR tests on nasopharyngeal swabs taken from 2 h to 16 days of newborns were negative in the current study.

## Conclusions

Our population-based cohort study in Wuhan shows that SARS-CoV-2 infection or diagnosis with COVID-19 during late pregnancy is associated with an increased risk of iatrogenic preterm birth and delivery with a cesarean section. In addition, our study has found little evidence to support maternal-fetal vertical transmission.

## Data Availability

The datasets for the current study are available from the corresponding authors on reasonable request.

## Abbreviations

COVID-19: Coronavirus disease 2019
CT: Computed tomography
MCHIMS: Maternal and Child Health Information Management System
MERS: Middle East respiratory syndrome
PROM: Premature rupture of membrane
SARS: Severe acute respiratory syndrome
SARS-CoV-2: Severe acute respiratory syndrome coronavirus 2
